# Spatial and Temporal Development of Müller Glial Cells in hiPSC-Derived Retinal Organoids Facilitates the Cell Enrichment and Transcriptome Analysis

**DOI:** 10.3389/fncel.2022.820396

**Published:** 2022-05-19

**Authors:** Rong Ning, Dandan Zheng, Bingbing Xie, Guanjie Gao, Jinhai Xu, Ping Xu, Yuan Wang, Fuhua Peng, Bin Jiang, Jian Ge, Xiufeng Zhong

**Affiliations:** ^1^State Key Laboratory of Ophthalmology, Zhongshan Ophthalmic Center, Sun Yat-sen University, Guangdong Provincial Key Laboratory of Ophthalmology and Visual Science, Guangzhou, China; ^2^Guangdong Provincial Key Laboratory of Brain Function and Disease, Faculty of Forensic Medicine, Zhongshan School of Medicine, Sun Yat-sen University, Guangzhou, China; ^3^Department of Neurology, The Third Affiliated Hospital of Sun Yat-Sen University, Guangzhou, China

**Keywords:** Müller glial cells, human induced pluripotent stem cells, retinal organoids, development, enrichment, transcriptome

## Abstract

Müller glial cells (MGCs) play important roles in human retina during physiological and pathological conditions. However, the development process of human MGCs *in vivo* remains unclear, and how to obtain large numbers of human MGCs with high quality faces technical challenges, which hinder the further study and application of MGCs. Human induced pluripotent stem cell (hiPSC)-derived retinal organoids (ROs) with all retinal cell subtypes provide an unlimited cell resource and a platform for the studies of retinal development and disorders. This study explored the development of human MGCs in hiPSC-derived ROs and developed an approach to select and expand the induced MGCs (iMGCs). In ROs, retinal progenitor cells progressively differentiated into SOX9+ Ki67– MGC precursors during differentiation day (D) 60 to D90, while mature MGCs expressing markers CRALBP and GS gradually appeared since D120, which spanned the entire thickness of the neural retina layer. Cells isolated from ROs aged older than 120 days was an optimal source for the enrichment of iMGCs with high purity and expansion ability. They had typical features of human MGCs in morphological, structural, molecular and functional aspects, and could be passaged serially at least 10 times, yielding large numbers of cells in a short period. The transcriptome pattern of the expanded iMGCs was also revealed. This study firstly clarified the timecourse of human MGC development in the RO model, where the iMGCs could be enriched and expanded, paving the way for downstream investigation and application in MGC-related retinal disorders.

## Introduction

Müller glial cells (MGCs), which are the principal glial component of vertebrate retina, originate along with retinal neurons from retinal progenitor cells (RPCs). It is reported that they are the last-born cell type during mouse retinal development (Dvoriantchikova et al., 2019). They span radially across the entire width of neural retina and constitute a structural scaffold to contact nearly all of retinal neurons (Bringmann et al., 2006; Eastlake et al., 2020). Due to this unique anatomical distribution, MGCs perform many vital physiological functions throughout the retina, including the formation of blood-retinal barrier, the control of extracellular space volume, ion and water homeostasis, and thus maintain the integrity of retina (Bringmann et al., 2006; Eastlake et al., 2020). They also provide neurotrophic, metabolic and anti-oxidative support for retinal neurons, and regulate the neuronal activity by neurotransmitters recycling, which are considered to be neuroprotective in nature (Reichenbach and Bringmann, 2013). These important characteristics of MGCs have generated scientific interests in their potential therapeutic applications, including as a vehicle to deliver benefit factors into the retina (Eastlake et al., 2020).

However, in pathological conditions, some studies indicate that MGCs can dedifferentiate into retinal progenitor-like cells to replenish injured neurons in zebrafish and chick (Raymond et al., 2006). This regenerative ability in the adult mammalian and human retina is still controversial. Many efforts have been made to identify the mechanism underlying regenerative potential of MGCs in order to establish new strategies for treatment of late-stage retinal diseases. In multiple types of human retinal diseases such as retinal detachment, proliferative vitreoretinopathy, diabetic retinopathy and retinal degenerative diseases, MGCs become activated by various pathogenic stimuli such as hypoxia and stress (Bringmann et al., 2006). The slight activation of MGCs may promote the survival of retinal neurons and so repair the retina, while the persistent activation may contribute to the formation of reactive gliosis and eventually lead to glial scar, accelerating the retinal neurodegeneration and greatly impeding the retinal repair (Bringmann et al., 2009; Bringmann and Wiedemann, 2012).

At present, most insights into MGCs are gained from experimental animal models and await confirmation on human cells. However, the pathophysiological mechanisms of human MGCs are still largely unknown due to the limited human cell source. To date, human MGCs are mostly obtained from cadaveric donors and samples after vitreoretinal surgery (Limb et al., 2002; Lawrence et al., 2007). In adult human retina, astrocytes, which share many features with MGCs, also present in retinal nerve fiber layer, and interfere the expansion and purification of MGCs *in vitro* (Lawrence et al., 2007). Moreover, immune rejection and risk of disease transmission are also obstacles of translational applications of these human MGCs (Eastlake et al., 2019). However, great progress in human induced pluripotent stem cells (hiPSCs) makes it possible to solve the above challenges. Retinal organoids (ROs), which can be generated by hiPSCs, are able to recapitulate the retinal development and form laminated retinal tissues (Zhong et al., 2014). Studies have demonstrated that the MGCs are the only glial cell type sharing a common progenitor with the retinal neurons, and they can emerge and survive in hiPSC-derived ROs (Zhong et al., 2014; Fligor et al., 2018; Capowski et al., 2019; Singh et al., 2021), providing many advantages in exploring the features of human MGCs.

Since there are many vital functions of MGCs in the human retina, the aim of our study is to clarify the developmental characteristics of human MGCs in hiPSC-derived ROs and establish protocols to enrich these cells, thus to facilitate the pathophysiological mechanism study of human MGCs and the treatment of MGC-related retinal diseases. Originating from RPCs, Ki67– SOX9+ CRALBP– GS– induced MGC (iMGC) precursors were identified in the early-stage ROs, while the Ki67– SOX9+ CRALBP+ GS+ mature iMGCs presented in the late-stage ROs. Importantly, the iMGCs were successfully enriched and expanded from the late-stage ROs, which exhibited similar morphological, structural, molecular and physiological features of primary human MGCs. The transcriptome pattern of the expanded iMGCs was also identified. Our findings provided new insights into the temporal and spatial development of human MGCs *in vivo* and established a simple approach to enrich these cells, laying the foundation for downstream investigation and application of human MGCs.

## Materials and Methods

### hiPSC Culture and RO Induction

Two hiPSC lines, BC1 and BC1-GFP lines were used in this study, which were gifts from Professor Linzhao Cheng (University of Science and Technology of China) (Chou et al., 2011; Zou et al., 2012). hiPSCs were cultured on Matrigel-coated (Corning, USA) plates in mTeSR1 medium (Stem Cell Technologies, Canada) and passaged at ~80% confluence every 5–7 days. RO induction was performed as previously described (Zhong et al., 2014; Li et al., 2018; Guan et al., 2021). Briefly, on differentiation day (D) 0, hiPSCs were dissociated and cultured in low adherent dishes to form embryoid bodies (EBs). EBs were plated on Matrigel-coated dishes during D5–D7 with neural induction medium (NIM), which was composed of DMEM/F12 (1:1), 1% N2 supplement (Invitrogen), 1% non-essential amino acids (NEAA) (Gibco) and 2 μg/ml heparin (Sigma-Aldrich). From D16, the culture medium was changed to retinal differentiation medium (RDM), containing DMEM/F12 (12:5), 2% B27 (without vitamin A, Invitrogen), 1% NEAA, and 1% antibiotic-antimycotic (Gibco). By week (W) 4–6, optic vesicles with neural retina (NR) domains turned up and were lifted up with tungsten needles, then cultured in suspension to spontaneously form ROs. Retinal culture medium (RCM), which comprised RDM, 10% fetal bovine serum (FBS) (Gibco), 100 μM Taurine (Sigma-Aldrich), and 2 mM GlutaMAX (Invitrogen) were used after 1 week of detachment. From D90, B27 supplemented in RCM was replaced by N2 for long-term RO culture. Medium was changed twice a week.

### Expansion Culture of iMGCs

NR layers were isolated from ROs at different timepoints (D60, D90, D120, and D150) after differentiation, dissected into small pieces with a pair of tungsten needles, and then dissociated into single cells by incubation of accutase (Gibco) for 15–25 min at 37°C. Afterwards, cell suspension was plated at a density of 1 × 10^4^ cells per cm^2^ on Matrigel-coated plates with MGC culture medium consisting of DMEM, 10% FBS, 2 mM GlutaMAX, 1% NEAA and 1% antibiotic-antimycotic. These cells were noted as passage 0 (P0). After cells were expanded, they were passaged at approximately 90% confluence every 6–10 days. Medium was changed every 3–4 days. MIO-M1, a commercial MGC line obtained from human retina (Limb et al., 2002; Lawrence et al., 2007), served as a control in designated experiments, was cultured and expanded at a density of 1 × 10^4^ cells per cm^2^ on Matrigel-coated plates in 1640 medium containing 10% FBS and 1% antibiotic-antimycotic. They were passaged at ~90% confluence every 4–6 days. Medium was changed every 2–3 days. The cell doubling time (DT) of the passaged cells were measured according to the following equation (Zou et al., 2019):


$$\begin{array}{l} DT=\text{t}\times \left[\mathrm{lg}2/\left(\mathrm{lg}N\text{t}-\mathrm{lg}N0\right)\right] \end{array}$$

where *N*_*t*_ represents the cell number at time period *t* (days) and *N*_0_ represents the initial cell number at the cell-plating day.

### Immunofluorescence Staining

Collected ROs were fixed in 4% paraformaldehyde (PFA, Sigma) for 30 min at room temperature. Mouse eyeballs were fixed in 4% PFA overnight at 4°C. Fixed ROs and mouse eyeballs were dehydrated through ascending grades of sucrose solutions from 6.25, 12.5, to 25%. Then they were embedded in O.C.T compound for frozen sections. Cells cultured on coverslips were fixed with 4% PFA for 8 min. Coverslips or sections were incubated in blocking solution consisting of 10% donkey serum and 0.25% Triton X-100 for 1 h at room temperature. Primary antibodies were incubated in the proper dilution overnight at 4°C. After that, the cells or sections were incubated with the corresponding secondary antibodies with either Alexa Fluor 488, 555, or 647 (Life Technologies, USA) for 1 h at room temperature. DAPI (4',6-diamidino-2-phenylindole, Dojindo Molecular Technologies, China) was used for counterstaining nuclei. The used primary antibodies are listed in Supplementary Table S1. Fluorescence images were acquired with a fluorescence microscope (Zeiss, Germany), a microscope slide scanner (Zeiss, Germany) or an LSM 880 confocal microscope (Zeiss, Germany).

### Scanning Electron Microscope

The passaged iMGCs were grown on coverslips and fixed in a mixture containing 2.5% glutaraldehyde and 2% PFA at 4°C overnight. Then these samples were sent to Electron Microscopy Core Facility at Fuda Testing Group (Guangzhou, China) for dehydration, drying, coating, and observed by scanning electron microscope (Apreo 2; FEI, Inc., Carlsbad, CA, USA).

### Transmission Electron Microscopy

The passaged iMGCs were cultured on plates and dissociated into cell suspension with 0.25% trypsin-EDTA (Gibco). Then cells were centrifuged into cell aggregates and fixed in mixture containing 2.5% glutaraldehyde and 2% PFA at 4°C overnight. After that, these samples were sent to Electron Microscopy Core Facility at Fuda Testing Group (Guangzhou, China) for dehydration, bedding, sectioning, staining and observed by transmission electron microscope (Tecnai G2 Spirit; FEI, Inc, Carlsbad, CA, USA).

### Electrophysiological Experiment

Whole-cell recordings at current-clamp mode were performed using an Integrated Patch-Clamp Amplifier (Sutter Instrument, USA) controlled by Igor 8 software (WaveMetrics, USA), filtered at 5 kHz and sampled at 20 kHz. Patch pipettes (4–6 MΩ) were filled with the internal solution containing the following (in mM): 130 K-gluconate, 10 HEPES, 10 KCl, biocytin 0.1–0.4%, 4 MgATP, 0.5 Na3GTP and 10 Na-phosphocreatine; pH 7.2–7.4. Osmolarity was adjusted to 290–300 mOsm. Recordings were performed at room temperature. Cultured iMGCs were perfused with ACSF (in mM): 124 NaCl, 3 KCl, 1.25 NaH_2_PO_4_, 1 MgCl_2_, 2 CaCl_2_, 26 NaHCO_3_, and 10 dextrose, bubbled with 95% O_2_/5% CO_2_. iMGCs were selected for study if they had a resting membrane potential <-30 mV. L-glutamate was applied to iMGCs by Picospritzer III (General Valve Corporation, USA) with Puff perfusion. The tip diameter of puff micropipettes was about 2–5 μm and puff pressure was between 4 and 6 psi. Glutamate at 100 μM was locally delivered using local pressure-puff.

### RNA-seq and Data Analysis

Samples from BC1-GFP hiPSCs and their derivatives were used to perform RNA-seq and data analysis. HiPSCs (~2 × 10^6^ cells per experiment, three independent experiments), NR layers isolated from D70-ROs and D150-ROs (five ROs per experiment, three independent experiments), P1 and P5 iMGCs from NR layers of D150-ROs (~1 × 10^6^ cells per experiment, three independent experiments) were collected in Trizol reagent (Invitrogen) and stored in a −80°C freezer. Then all of these samples were submitted to Gene Denovo Biotechnology Co. (Guangzhou, China) for RNA extraction, library construction, sequencing and data analyses. Total RNA was extracted using Trizol reagent kit (Invitrogen) according to the manufacturer's protocol. RNA quality was assessed on an Agilent 2100 Bioanalyzer (Agilent Technologies). Oligo (dT) beads were used to isolate the poly mRNA from the total RNA. The enriched mRNA was fragmented and reverse transcribed into cDNA using random primers. After synthesis of the second strand, the cDNA was purified, end-repaired and ligated to Illumina sequencing adapters. The ligation products were size selected, amplified, and sequenced. Raw reads were filtered and the clean reads were obtained. Afterwards, an index of the reference genome was built, and paired-end clean reads were mapped to the reference genome using HISAT2 (Kim et al., 2015). The mapped reads of each sample were assembled by using StringTie (Pertea et al., 2016). For each transcription region, a fragment per kilobase of transcript per million mapped reads (FPKM) value was calculated to quantify its expression abundance and variations, using RSEM software (Li and Dewey, 2011). Correlation analysis was performed by R. Correlation and principal component analysis (PCA) was performed with R package gmodels (http://www.r-project.org/). Differential expression analysis was performed by DESeq2 software (Love et al., 2014) between two different groups. The genes/transcripts with the parameter of false discovery rate (FDR) below 0.05 and absolute fold change ≥2 were considered differentially expressed genes/transcripts (DEGs). Then the upregulated DEGs were underwent Gene Ontology (GO) and Kyoto Encyclopedia of Genes and Genomes (KEGG) pathway enrichment analysis, and the GO terms or pathways with an adjust *p*-value ≤ 0.05 (qvalue) were defined as significant enriched GO terms or pathways.

### Animals and Subretinal Transplantation of iMGCs

NOD/SCID mice were used in this study. The animal study was reviewed and approved by the Animal Ethics Committee of the Zhongshan Ophthalmic Center, Sun Yat-Sen University. All experiments complied with national animal care guidelines. Four to six-week-old NOD/SCID mice were divided into two groups: a treatment group injected with the P2 or P5 iMGCs from BC1-GFP or BC1 ROs older than D120 (1 ×10^5^ cells in 1.5 μl DMEM per eye), and a vehicle control group injected with DMEM (1.5 μl per eye). Subretinal injections were performed using a 2.5 μl Hamilton syringe and 33 G needle (Hamilton, Swizerland). Fundus photography (Topcon TCR-50DX) and optical coherence tomography (OCT, Envisu R-Class) examination were done to dynamically track the donor cells in subretinal space (SRS). These transplanted mice were monitored for about 4 weeks. The mice were sacrificed by cervical dislocation at days 3, 7, and 21 after surgery. The experimental eyeballs of mice were removed and fixed with 4% PFA overnight at 4°C.

### Statistical Analysis

Statistical analyses were performed using GraphPad Prism Software 8.0.1 (GraphPad Software, USA). The results of electrophysiological experiment are presented as the mean ± SEM (standard error of mean), and other results are presented as the mean ± SD (Standard Deviation). *P* < 0.05 was considered statistically significant. For statistical analysis of RNA-seq, see RNA-seq and Data Analysis for more details.

## Results

### Temporal and Spatial Development of MGCs in hiPSC-Derived ROs

**Figure 1:**
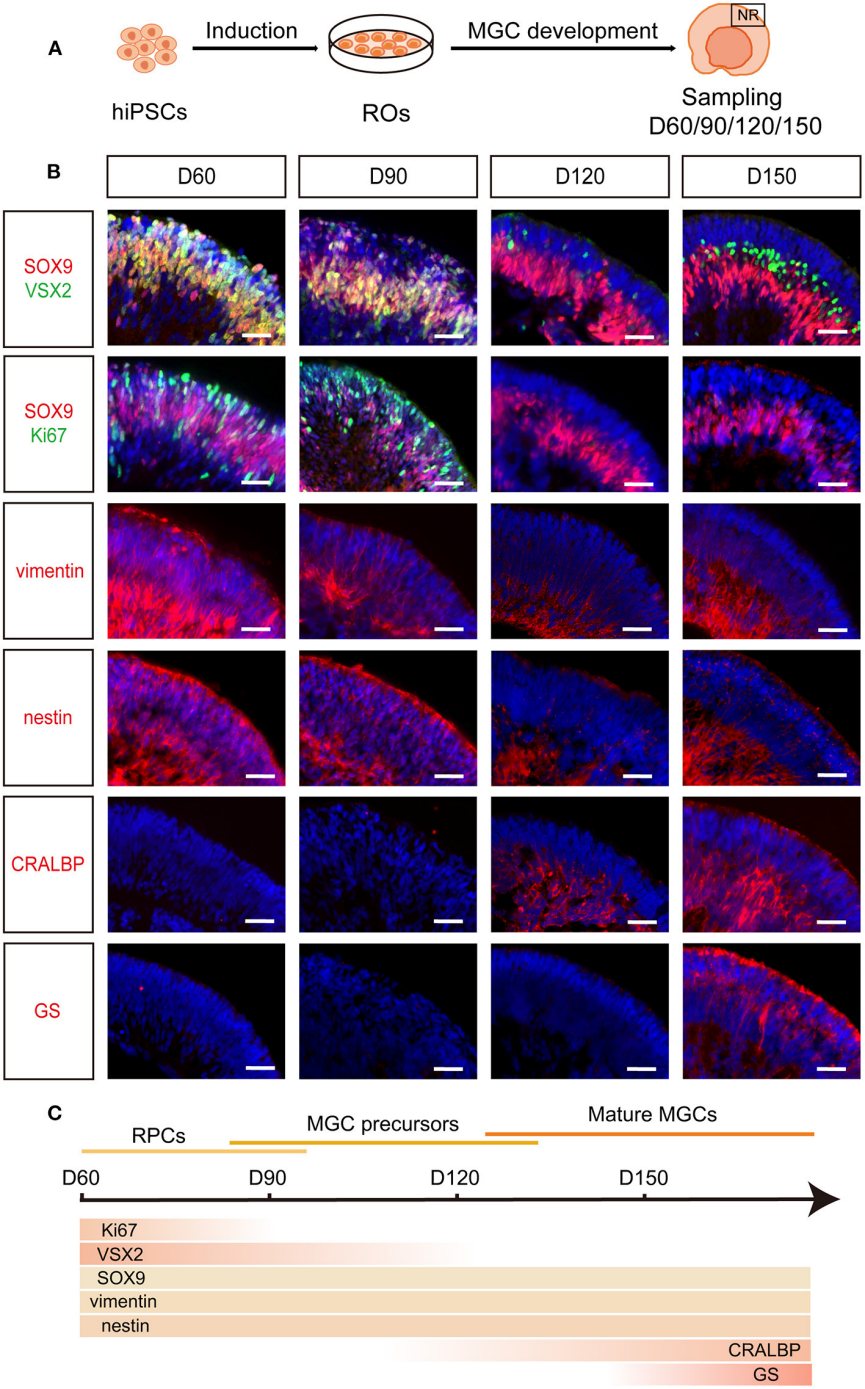
Spatiotemporal development of MGCs in hiPSC-derived ROs. **(A)** Schematic overview of the development of MGCs in ROs. **(B)** Immunofluorescence staining showed the expression of markers VSX2, SOX9, Ki67, vimentin, nestin, CRALBP and GS in D60-, D90-, D120-, and D150-ROs. **(C)** Schematic diagram of MGCs development in hiPSC-derived ROs. hiPSC, human induced pluripotent stem cell; ROs, retinal organoids; MGC, Müller glial cell; NR, neural retina. Scale bars = 50 μm **(B)**.

To observe the development of human MGCs, dissociated hiPSCs were differentiated into ROs according to our published retinal differentiation protocol (Zhong et al., 2014; Li et al., 2018; Guan et al., 2021). During W4–W7, three-dimensional (3D) ROs with thick and transparent NRs spontaneously formed, resembling human eye-cups (Figure 1A; Supplementary Figure S1). Immunofluorescence staining of these ROs showed that at the early stage of differentiation (D60–D90), most cells in the NR layer firstly co-expressed RPC markers Ki67, SOX9, VSX2, vimentin and nestin, representing a population of RPCs (Figures 1B,C). Some early-born retinal neurons such as BRN3+ PAX6+ ganglion cells and CRX+ photoreceptor cells were also seen in the NR layer at this stage (Supplementary Figures S2A–C). As the differentiation progressed, RPCs exited the cell cycle and differentiated into Ki67– VSX2– SOX9+ CRALBP- GS- cells, which gradually migrated to the intermediate layer of NRs and were defined as MGC precursors to represent a transitional period of developing MGCs between RPCs and mature MGCs (Figures 1B,C). At the late stage of differentiation (D120 afterwards), the MGCs expressed markers CRALBP and GS specific for mature MGCs (Limb et al., 2002; Eastlake et al., 2019) and stretched the whole width of NRs with their cell processes (Figures 1B,C; Supplementary Figures S3A,B). RPC and MGC markers SOX9, nestin and vimentin were also expressed in the intermediate layer of NRs from D120 (Figures 1B,C), implying the mature MGCs emerged in the late-stage RO (Zhong et al., 2014; Fligor et al., 2018; Capowski et al., 2019). Surprisingly, GFAP, a marker for astrocyte and active MGC (Vecino et al., 2016; Reichenbach and Bringmann, 2020), was not expressed in the NR of ROs at both the early and late stages (Supplementary Figures S2D, S4). In addition, the NR layers were negative for optic stalk marker PAX2 and forebrain cell marker SOX1, further confirming MGCs in NR layers indeed originated from retina (Supplementary Figure S2D).

**Figure 2:**
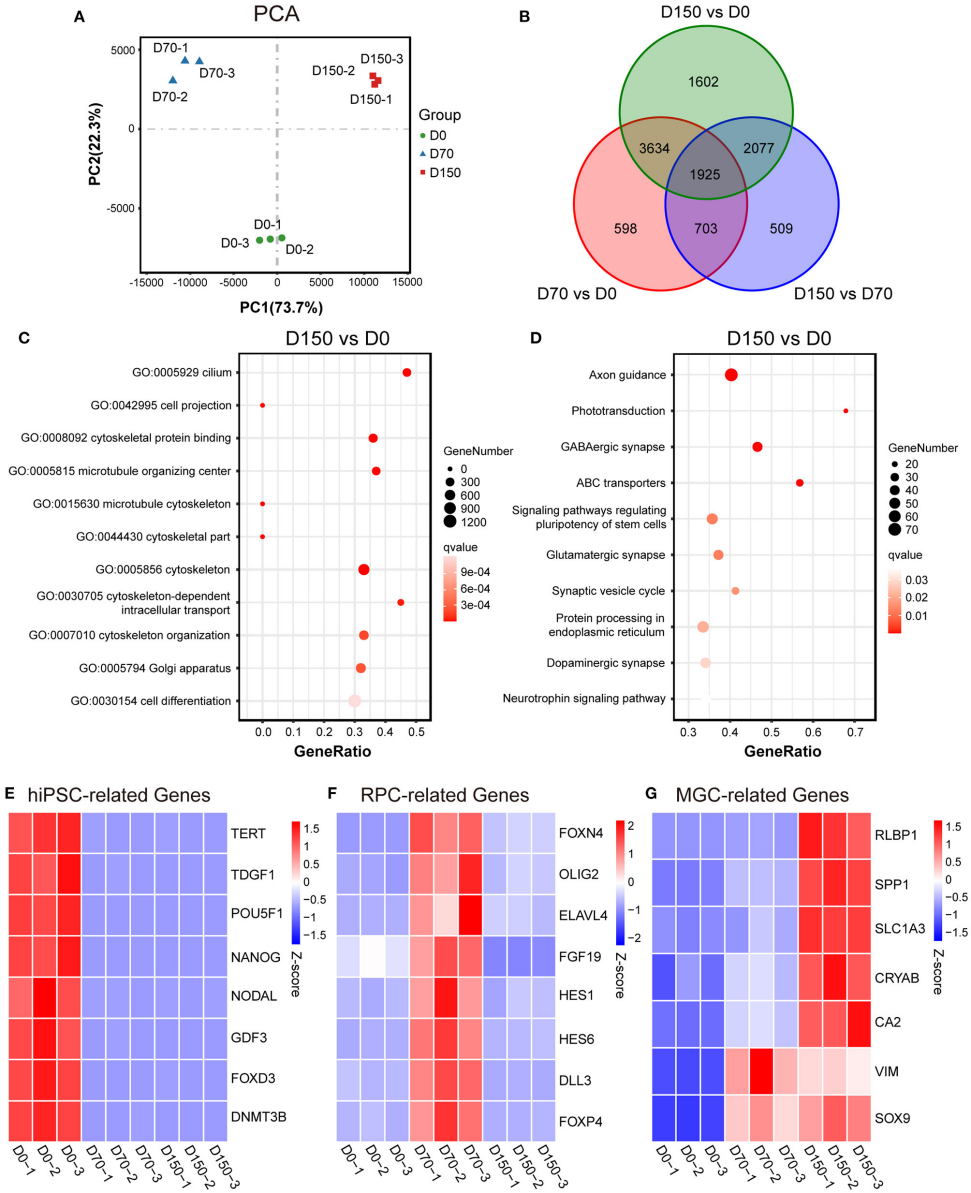
RNA-seq analysis of MGC development in ROs at different stages. **(A)** Principal components analysis (PCA) showed a comparison of the transcriptome data in hiPSCs (D0) and ROs at different stages. **(B)** Venn diagram showed the number and overlapping relations of the differentially expressed genes (DEGs) among hiPSCs and ROs at different stages. **(C)** Dot plot showed Gene Ontology (GO) analysis of the upregulated DEGs of D150-ROs comparing to hiPSCs; GO terms with an adjust *p*-value (qvalue) ≤ 0.05 were defined as significant enriched GO terms. **(D)** Dot plot showed Kyoto Encyclopedia of Genes and Genomes (KEGG) analysis of the upregulated DEGs of D150-ROs comparing to hiPSCs; signaling pathways with qvalue ≤ 0.05 were defined as significant enriched pathways. **(E–G)** Heatmap showed the expression pattern of the hiPSC-related genes **(E)**, RPC-related genes **(F)** and MGC-related genes **(G)** in hiPSCs (D0), D70-ROs (D70), and D150-ROs (D150). Blue to red indicated a gradient from low to high gene expression. hiPSC, human induced pluripotent stem cell; NR, neural retina; ROs, retinal organoids; RPC, retinal progenitor cell; MGC, Müller glial cell; −1, −2, and −3, experimental replicates; VS, versus. *VIM*, gene name of vimentin; *RLBP1*, gene name of CRALBP.

RNA-seq analysis further characterized the development of MGCs during retinal differentiation. PCA indicated developmental age as the first principal component explaining almost 74% of the variance (Figure 2A). Differential expression analysis showed that there were substantial differences among hiPSCs and ROs at different stages (Figure 2B; Supplementary Figures S5A,B). When compared with hiPSCs, the number of differential expression genes (DEGs) that showed more than or equal to 2-fold change in D70-ROs or D150-ROs was, respectively, up to 6860 and 9238. And when compared with D70-ROs, there were 5214 DEGs in D150-ROs (Figure 2B; Supplementary Figures S5A,B). GO analysis showed that the upregulated DEGs of D150-ROs comparing to hiPSCs were enriched in cellular components related to MGCs development, such as cytoskeleton, cilium, cell projection (Figure 2C). While KEGG analysis revealed that the upregulated DEGs of D150-ROs comparing to hiPSCs were enriched in signaling pathways related to the functions of MGCs, such as Glutamatergic synapse (Figure 2D). Pluripotency marker genes including *FOXD3, TERT, TDGF1*, and *NANOG* were high expressed in hiPSCs but low in hiPSC-derived ROs (Figure 2E). In addition, RPC-related genes such as *OLIG2 and DLL3* were enriched in the early-stage ROs (Figure 2F). While MGC-related genes, such as *CRYAB, RLBP1, CA2*, and *SLC1A3* were progressively upregulated over the differentiation process (Figure 2G; Supplementary Figure S5C), which were consistent with the previous studies (Hoshino et al., 2017; Kim et al., 2019; Yan et al., 2020; Couturier et al., 2021). The expression of RPC and MGC genes *SOX9* and *VIM* significantly increased in the early-stage ROs when compared to hiPSCs and kept high expression level in late-stage ROs (Figure 2G). Additionally, the expression of astrocyte marker *GFAP* and forebrain cell marker *SOX1* were not detectable, and the expression level of the optic stalk marker *PAX2* was very low in ROs at different stages (Supplementary Figure S5C). Part of these results were verified by the immunostaining analysis described above (Figure 1B; Supplementary Figures S2D, S4).

Altogether, RPCs from both hiPSC lines sequentially differentiated into MGC precursors and mature MGCs in hiPSC-derived ROs and the mature MGCs were spatially located in the intermediate layer of NRs, which recapitulates the spatiotemporal pattern of MGC development in human fetal retina (Figure 1C) (Xiang, 2013; Quinn and Wijnholds, 2019).

### The Enrichment of iMGCs From hiPSC-Derived ROs

**Figure 3:**
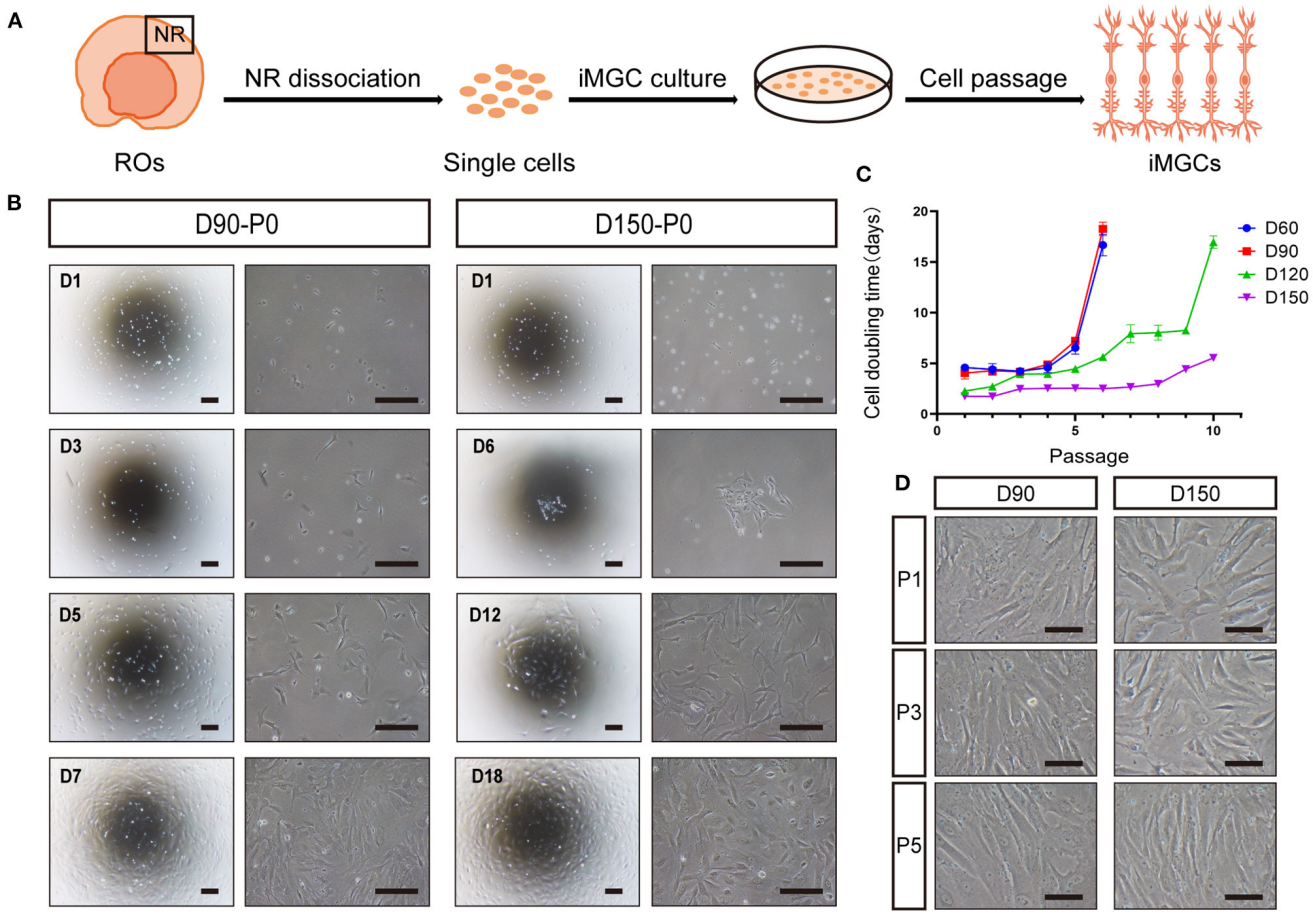
Optimal expansion of induced MGCs (iMGCs) from hiPSC-derived ROs. **(A)** Schematic diagram of expansion of iMGCs from hiPSC-derived ROs. **(B)** Bright-field images showed the distinguished morphological features of the passage 0 (P0) cells isolated from D90-ROs (D90-P0) and D150-ROs (D150-P0). **(C)** The cell doubling time showed different expansion capability of cells isolated from D60-, D90-, D120-, and D150-ROs. **(D)** Bright-field images showed no obvious morphological difference in P1, P3, and P5 cells expanded from D90- and D150-ROs, all appearing a spindle-like shape. ROs, retinal organoids; NR, neural retina. Scale bars = 200 μm **(B)** and 100 μm **(D)**.

To acquire MGCs with high purity and expansion ability, according to the timecourse of MGC development in ROs described above, we tried to expand the MGCs from ROs at early (D60, D90) and late (D120, D150) stages. NR layers isolated from ROs were dissociated into retinal cells, and these cells were seeded in culture plates, noted as P0 (Figure 3A). The primary cells from early- and late-stage ROs are both expandable, but exhibited different growth patterns (Figure 3B). As for cells from the early-stage ROs, majority of primary cells survived, proliferated quickly and reached confluency in about 1 week (Figure 3B). In contrast, only a few of primary cells from the late-stage ROs could survive, grew in a single clone, proliferated slowly and took more than 2 weeks to reach confluency (Figure 3B). In addition, the cell doubling time of the passaged cells peaked at P6 cells from the early stage ROs and P10 ones from the late-stage ROs, respectively (Figure 3C), indicating that cells from the latter could expand more times than from the former. After passage, the expanded cells from both the early- and late-stage ROs were progressively elongated and exhibited a spindle-like shape with rough membrane and abundant projections in a few days (Figure 3D), showing similar morphological features of primary MGCs isolated from human adult retina (Limb et al., 2002; Lawrence et al., 2007).

**Figure 4:**
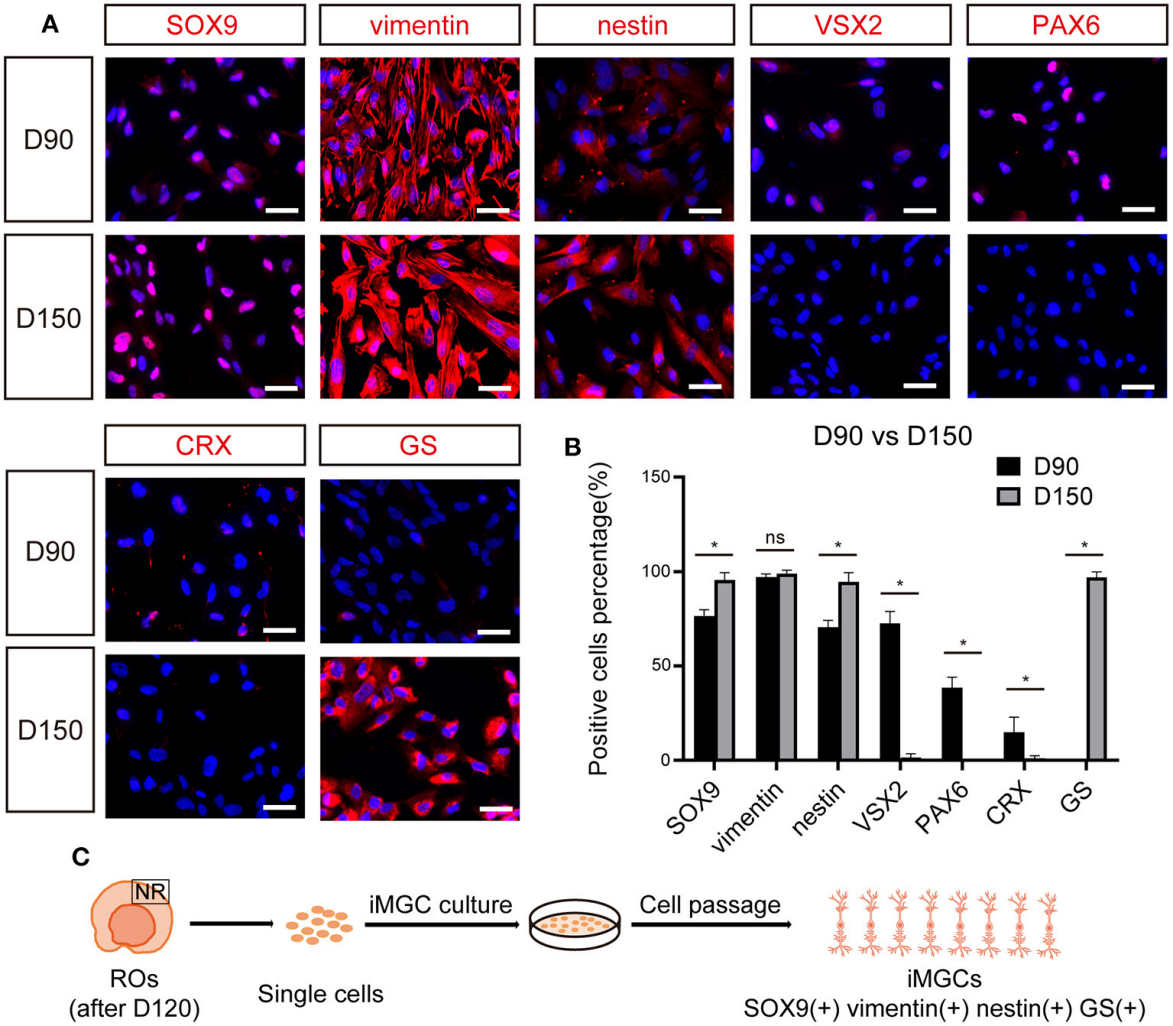
The expression of cell-specific markers in passaged iMGCs from early- and late- stage ROs. **(A)** Immunofluorescence staining showed the expression of markers SOX9, vimentin, nestin, VSX2, PAX6, CRX, and GS in the passaged cells isolated from D90- and D150-ROs. **(B)** Percentage of positive cells in **(A)**. Cell number > 100, *p < 0.05. **(C)** Schematic diagram of molecular characteristics of iMGCs isolated from the late-stage ROs. ROs, retinal organoids; ns, no significance; Scale bars = 50 μm **(A)**.

Immunofluorescence staining was performed to validate the molecular signature of the passaged cells from ROs at different stages. The majority of passaged cells from early-stage ROs strongly expressed RPC and MGC markers SOX9, vimentin and nestin, but did not express key mature MGC marker GS (Figures 4A,B). Some of these cells weakly expressed VSX2 and PAX6 and a few of them expressed photoreceptor cell marker CRX (Figures 4A,B). These results indicated that the passaged cells from early-stage ROs might be mixtures of RPCs, MGC precursors and the other retinal neurons. Whereas, nearly all passaged cells from late-stage ROs were positive for MGCs markers SOX9, vimentin, nestin and GS, but rarely expressed markers VSX2, PAX6, or CRX, indicating that these cells were MGCs with high purity (Figures 4A–C; Supplementary Figure S6A).

Collectively, above data indicated that the late-stage ROs were the optimal choice for the enrichment of iMGCs which could be serially expanded more than 10 passages, yielding large numbers of cells with high quality and purity. For example, 10 late-stage ROs could produce ~100 million P2 cells within 15 days. These cells could be also cryopreserved for future applications. The P1 to P5 iMGCs were used for further characterization in this study unless otherwise noted. In addition, although the morphological and molecular features of the expanded iMGCs from the BC1 and BC1-GFP hiPSCs tested were quite similar, the variability of iMGCs from these two lines were also observed. The iMGCs from BC1-GFP ROs had a slightly stronger expansion ability than those from BC1 ROs at the same developmental stage.

### Transcriptome Analysis and Phenotype Stability of the Passaged iMGCs

**Figure 5:**
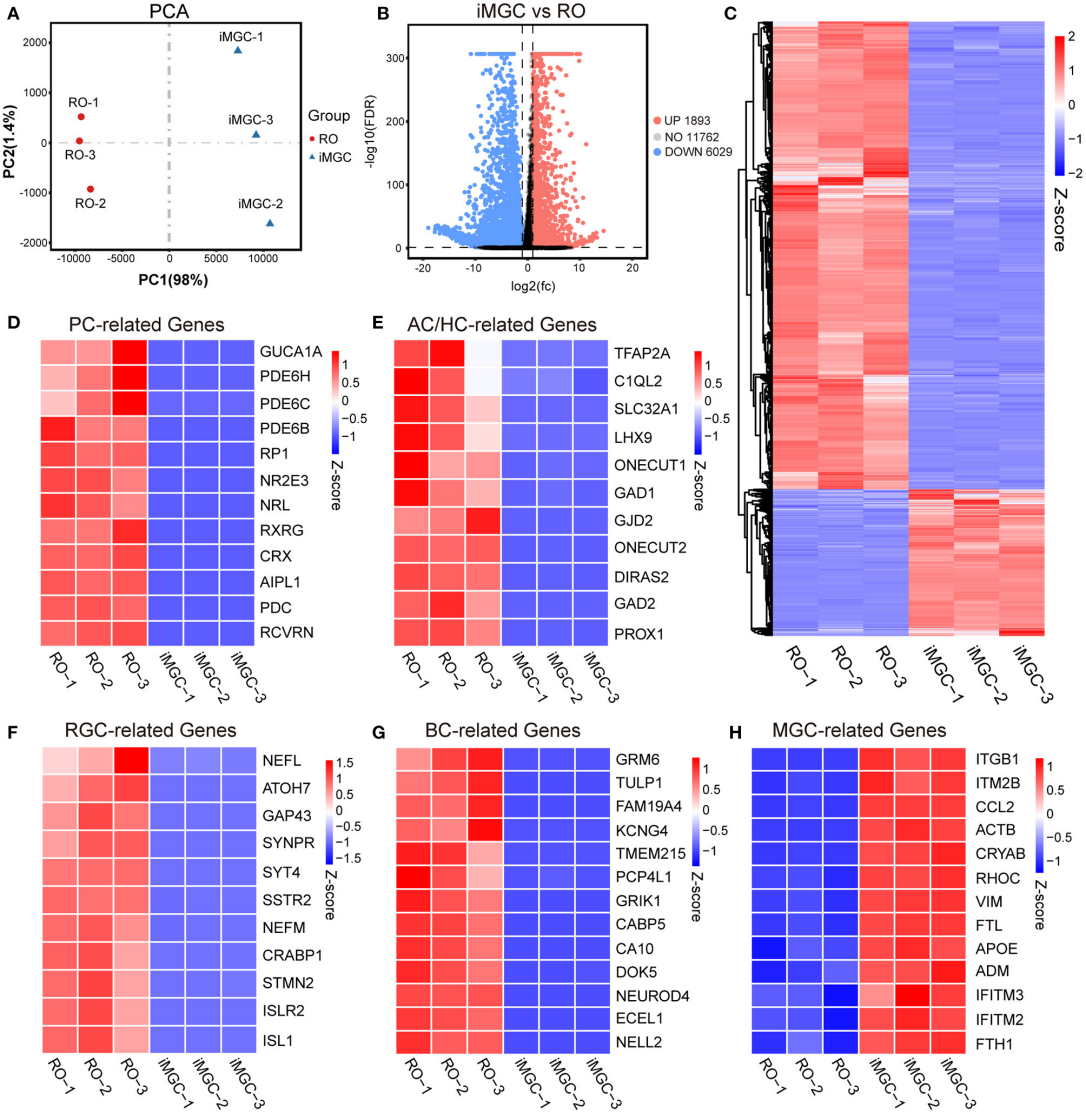
The transcriptome signatures of the passaged iMGCs. **(A)** Principal components analysis (PCA) showed a comparison of the transcriptome data in the passaged iMGCs and D150-ROs. **(B)** Volcano plot showed the total number of the upregulated (UP) and downregulated (DOWN) differentially expressed genes (DEGs) between the NR layers and the passaged iMGCs. **(C)** Heatmap showed the total DEGs between the NR layers and the passaged iMGCs. **(D–H)** Heatmap exhibited the expression of the photoreceptor-related (PC-related) genes **(D)**, amacrine and horizontal cell-related (AC/HC-related) genes **(E)**, retinal ganglion cell-related (RGC-related) genes **(F)**, bipolar cell-related (BC-related) genes **(G)**, and Müller glial cell-related (MGC-related) genes **(H)** between the NR layers and the passaged iMGCs. Blue to red indicated a gradient from low to high gene expression. ROs, retinal organoids; NR, neural retina; FDR, false discovery rate; FC, fold change; iMGC, the passaged iMGCs isolated from D150-ROs; RO, NR layers isolated from D150-ROs; −1, −2, and −3: experimental replicates; NO, no significance; VS, versus.

Next, we examined the transcriptomes of the passaged iMGCs from the late-stage ROs. RNA-seq analysis showed the global difference of transcriptomes between ROs and the iMGCs (Figures 5A–C). Photoreceptor-related genes such as *CRX* and *RCVRN*, amacrine and horizontal cell-related genes such as *PROX1* and *TFAP2A*, retinal ganglion cell-related genes such as *ATOH7* and *ISL1*, and bipolar cell-related genes such as *GRM6* and *CA10*, were downregulated in the iMGCs, but up-regulated in D150 ROs. While the expression of MGC-related genes such as *VIM, CRYAB, APOE*, and *CCL2* (Hoshino et al., 2017; Kim et al., 2019; Yan et al., 2020; Couturier et al., 2021) were enriched in the passaged iMGCs, but down-regulated in D150 ROs (Figures 5D–H). Moreover, all of these passaged iMGCs expressed neither GFAP protein (Supplementary Figure S7), nor *GFAP, SOD3, S100B, PAX2, CSF1R*, or *CX3CR1* mRNA (FPKM <1, data not shown), indicating there were no contamination of non-retinal cells or tissue such as astrocyte, optic stalk and microglia in these passaged iMGCs (Westergard and Rothstein, 2020; Bosze et al., 2021). Therefore, RNA-seq data further confirmed that the iMGCs from the late-stage ROs were enriched with typical transcriptome signature, and not contaminated with other retinal subtypes or non-retinal cells.

We further analyzed the phenotype stability of the iMGCs after serial passages. Immunofluorescence staining showed that there were no significant differences in the expression of specific markers between P1 and P5 iMGCs (Supplementary Figure S6A). Both the P1 and P5 iMGCs abundantly expressed MGC markers SOX9 and GS, but rarely expressed markers VSX2, PAX6 or CRX for other retinal subtypes (Supplementary Figure S6A). Surprisingly, the MGC marker CRALBP became negative from passage 1 (Supplementary Figure S6A), which was consistent with a previous study on MGCs isolated from ROs (Couturier et al., 2021). RNA-seq analysis illustrated that the majority of genes including the classical MGC marker genes such as *SOX9, VIM, GLUL, APOE*, and *SPP1* presented similar transcript level between the P1 and P5 iMGCs, demonstrating the phenotype stability of the iMGCs after several passages (Supplementary Figures S6B–D).

Remarkably, when compared to MIO-M1, a spontaneously immortalized MGC line obtained from human retina (Limb et al., 2002), iMGCs were much larger in size with a long, spindle-like shape and abundant cytoplasm, and expressed only the MGC markers SOX9, vimentin, nestin and GS. While some MIO-M1 cells expressed not only the MGC markers, but also markers such as VSX2, PAX6, and CRX for other retinal subtypes and GFAP for astrocytes (Supplementary Figures S7A,B). These findings implied that long-term expanded MIO-M1 over 65 passages might progressively lose the molecular and morphological signatures of human MGCs under 2D culture conditions. Altogether, iMGCs expanded from hiPSC-derived ROs did not contaminate astrocytes, and maintained the typical features of primary human MGCs within several passages.

### The Ultrastructural and Functional Characteristics of iMGCs

**Figure 6:**
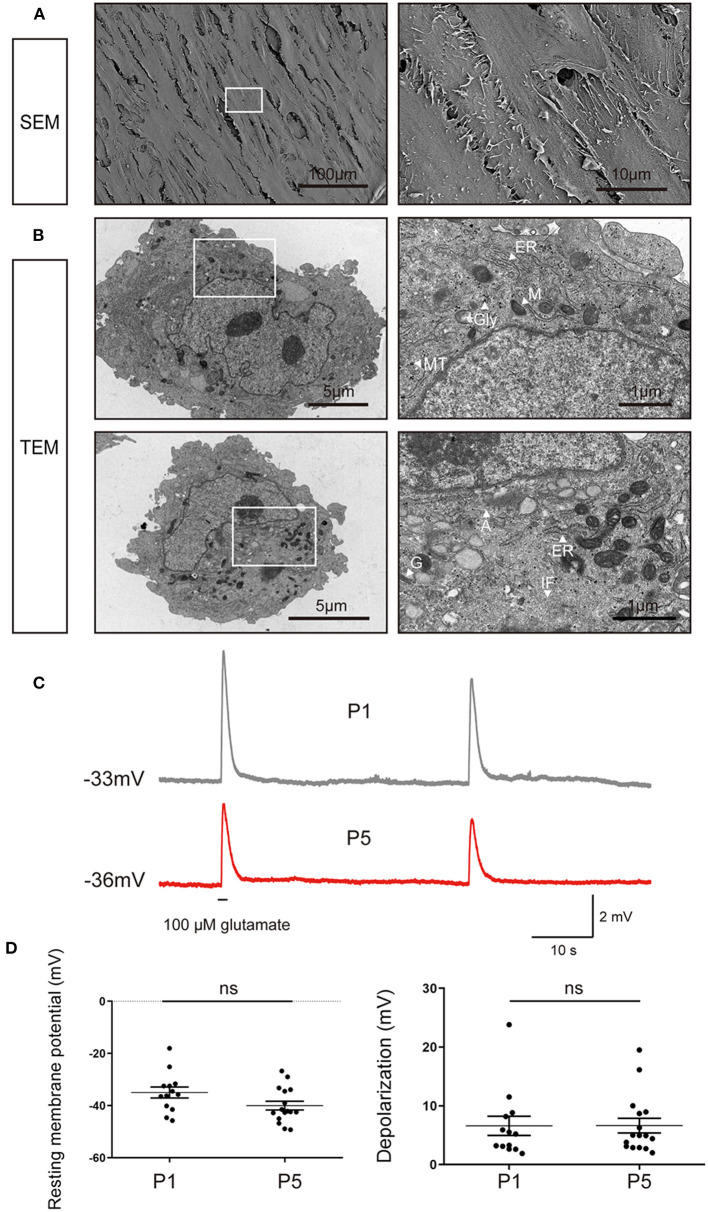
Ultrastructural and electrophysiological characteristics of the passaged iMGCs. **(A)** SEM images showed the iMGCs possessed a typical spindle-like morphology with many microvillus. **(B)** TEM images showed the iMGCs with abundant mitochondria (M), endoplasmic reticulum (ER), Golgi apparatus (G), glycogen (Gly), microtubules (MT), intermediate filaments (IF) and actins (A) in the cytoplasm. **(C)** Both P1 and P5 iMGCs depolarized after application of L-glutamate in current-clamp recordings. **(D)** P1 and P5 iMGCs presented no significant difference in resting membrane potential and the magnitude of depolarization. SEM, scanning electron microscope; TEM, transmission electron microscope; P1 and P5, passage 1 and 5; ns, no significance.

To further characterize the structural signatures of the passaged iMGCs, SEM and TEM were used to observe the ultrastructure of the passaged iMGCs (Figures 6A,B). The cells displayed a spindle-like morphology with microvillus projections and contained large nucleus with obvious nucleoli. In these iMGCs, Golgi apparatus, mitochondria, endoplasmic reticulum surrounded the nucleus. Abundant glycogen particles and cytoskeletal elements including microtubules, intermediate filaments and actin filaments presented in cytoplasm (Figures 6A,B), which were consistent with the features of MGCs isolated from human retina (Limb et al., 2002; Lawrence et al., 2007).

Then membrane potential of iMGCs were recorded in response to L-glutamate puff. The electrophysiological properties of these iMGCs were similar to MGCs isolated from human retina (Limb et al., 2002; Lawrence et al., 2007) (Figure 6C). At current-clamp mode, the membrane potential depolarized from −34.97 to ~ −28.38 mV (6.59 ± 1.64 mV change, resting membrane potential −34.97 ± 2.10 mV, *N* = 13 cells) in P1 iMGCs, and from −40.01 to −33.37 mV (6.64 ± 1.29 mV change, resting membrane potential −40.01 ± 1.74 mV, *N* = 16 cells) in P5 iMGCs (Figure 6C). There was no significant difference in resting membrane potential and the magnitude of depolarization induced by L-glutamate between P1 and P5 iMGCs (Figure 6D, *p* > 0.05, unpaired *t*-test), suggesting the iMGCs still maintained the functional characteristics of human MGCs even after several passages.

### Evaluation of Cell Survival of the iMGCs After Subretinal Transplantation Into Mice

**Figure 7:**
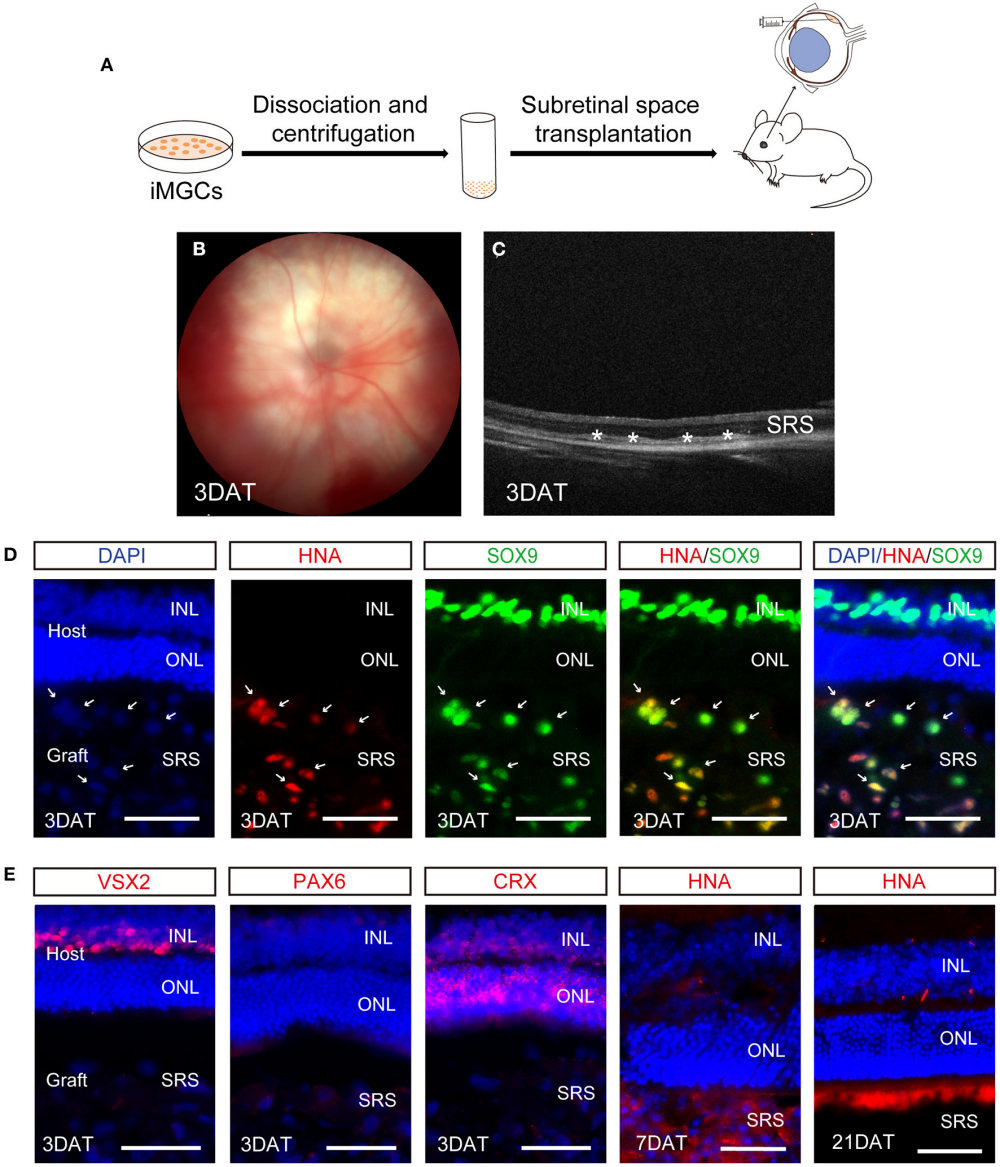
Evaluation of cell survival of the passaged iMGCs after subretinal transplantation into NOD/SCID mice. **(A)** Schematic overview of the subretinal transplantation process of the iMGCs. **(B,C)** Fundus photography **(B)** and optical coherence tomography (OCT) **(C)** showed the successful delivery of the iMGCs into subreinal space (SRS) in NOD/SCID mice 3 days after transplantation (DAT). *The grafted cells. **(D,E)** Immunofluorescence staining showed the grafted iMGCs (D3, P2 iMGCs from D120 BC1-ROs) were positive for HNA and SOX9, but negative for VSX2, PAX6, and CRX at 3 DAT, and gradually disappeared from 7 to 21 DAT. INL, inner nuclear layer; ONL, outer nuclear layer. Scale bars = 50 μm **(D,E)**.

Although studies have shown that MGCs can serve as endogenous stem cells to regenerate neural retina in zebrafish and chick (Fischer and Reh, 2001; Raymond et al., 2006), this regenerative capacity remain debatable in mammalian and human retina. Here, we performed a preliminary evaluation about the cell survival of the iMGCs in subretinal space of NOD/SCID mice (Figure 7A). A total of 19 eyes were injected with iMGCs, followed by 21-day observations. During the observation period, all mice kept healthy and no tumors were observed. Fundus photography and OCT examination showed the successful delivery of cells with high reflection located in the subretinal space (SRS) in 3 days after transplantation (DAT) (Figures 7B,C). Immunofluorescence staining confirmed that the substantial grafted cells survived in 3 DAT, and co-expressed MGC marker SOX9 and human nucleus antigen marker (HNA), but were negative for other retinal neuron markers VSX2, PAX6, or CRX (Figures 7D,E). However, the grafted cells gradually deteriorated since 7 DAT and almost disappeared in SRS at 21 DAT (Figure 7E). During the observation period, the donor cells rarely migrated into the host retina of mice. The above data indicated that the iMGCs could not survive well and transdifferentiate into other retinal neurons after transplantation.

## Discussion

In this study, we firstly clarified the spatiotemporal development of human MGCs in a hiPSC-derived RO model and developed a simple approach to enrich human MGCs from ROs. MGC precursors and mature MGCs emerged sequentially from the early to the late-stage ROs, where they spatially spanned the entire thickness of NR with their soma located in the intermediate layer of NR. These observations were consistent with the developmental timecourse and the spatial characteristics of MGCs in human retina (Ohsawa and Kageyama, 2008; Kohwi and Doe, 2013; Xiang, 2013; Quinn and Wijnholds, 2019; Singh et al., 2021). Importantly, the optimal timepoint of ROs to enrich MGCs were explored. Although cells isolated from both early- and late-stage ROs were expandable, the late-stage ROs were the most optimal option for enriching iMGCs with high purity and long-term expansion ability. They possessed morphological, structural, molecular and functional characteristics of primary human MGCs, had the ability to serially expand for at least 10 passages, producing a large population of iMGCs in a short period. The transcriptome pattern of the expanded iMGCs was also revealed. Surprisingly, the passaged iMGCs survived only a short-term period and did not transdifferentiate into other types of retinal cells after transplantation into NOD/SCID mice. Altogether, this study has revealed the developmental and biological features of human MGCs and provided a valuable cell source, which would significantly promote the investigation and application in MGC-related retinal disorders.

Studies in animal models and cell lines show that the retinal glia differentiates from a progenitor-like state, via a stage of immature glia, and finally to mature MGCs (Ohsawa and Kageyama, 2008; Kohwi and Doe, 2013; Xiang, 2013; Dvoriantchikova et al., 2019; Quinn and Wijnholds, 2019). These differentiation progresses involve morphological, biochemical and physiological changes (Ohsawa and Kageyama, 2008; Kohwi and Doe, 2013; Xiang, 2013; Dvoriantchikova et al., 2019; Quinn and Wijnholds, 2019). However, there are few reports referring to the marker sets which can be used to define immature MGCs or MGC precursors. In this study, RPCs appeared at the early stage of ROs, co-expressing markers Ki67, VSX2, and SOX9; while at D90 ROs, SOX9+ VSX2– or SOX9+ Ki67– cells began to appear in the NR layer, which lost the characteristics of RPCs but did not express mature MGC markers CRALBP or GS. Since D120, Ki67– SOX9+ VSX2– CRALBP+ GS + mature MGCs were clearly identified in the NR layer. Therefore, we defined the Ki67– SOX9+ CRALBP– GS– cells as MGC precursors to represent a transitional period of developing MGCs between RPCs and mature MGCs. These MGC precursors eventually differentiated into mature MGCs, sufficiently expressing CRALBP and GS, which is consistent with the fact that mature MGCs accumulate glia-specific protein and enzymes such as CRALBP and GS (Limb et al., 2002; Lawrence et al., 2007). RNA-seq analysis also revealed that from the early to the late stage ROs, RPC-related genes were downregulated, while MGC-related genes were continuously upregulated, indicating that RPCs progressively obtained the MGC fate as differentiation progressed. In addition, the unique morphology and special distribution of MGCs are associated with many vital physiological functions. For example, they provide an orientation scaffold and migration substrate for post-mitotic young neurons (Reichenbach and Bringmann, 2013, 2020). In hiPSC-derived ROs, the mature MGCs radially spanned the entire thickness of NR with their soma located in the intermediate layer of NR, which is consistent to the morphological features of MGCs in human retina (Reichenbach and Bringmann, 2020). The detailed elucidation of the temporal and spatial development of iMGCs in hiPSC-derived ROs greatly promotes the understanding of human MGC development *in vivo*.

MGCs possess plenty of significant functions in the retinal pathological conditions, but the functional mechanisms are still largely unknown. Enrichment of human MGCs *in vitro* is of great significance for further MGC mechanism studies, which may help to develop effective therapeutic targets for the treatment of various MGC-related human retinal disorders. However, at present, human MGCs are mostly obtained from cadaveric donors and samples after vitreoretinal surgery (Limb et al., 2002; Lawrence et al., 2007), which is limited by scarce donor, astrocyte contamination, immune rejection and risk of disease transmission (Eastlake et al., 2019). There are also some spontaneously immortalized MGC lines obtained from human retina, such as MIO-M1 (Limb et al., 2002; Lawrence et al., 2007). However, in this study, we found that after too many serial passages, their molecular and morphological signatures have altered progressively and there was still astrocyte contamination among these cells. hiPSC-derived ROs containing MGCs can serve as an unlimited source for the selection and enrichment of human MGCs without immune rejection and risk of disease transmission. Moreover, we noticed that the optic stalk marker PAX2, forebrain cell marker SOX1 and astrocyte marker GFAP were negative in the NRs of late-stage ROs in our differentiation system, which could exclude the contamination of non-retinal cells in the subsequent MGCs isolation. Therefore, ROs have advantages to provide purer MGCs than retinal tissues for downstream studies, solving an issue existing in the field for long-term.

A couple of groups have tried to expand iMGCs from ROs at different developmental stages ranged from D34 to D281 (Chung et al., 2019; Eastlake et al., 2019; Couturier et al., 2021). However, the optimal stage of ROs for enrichment of iMGCs has not been discussed. In this study, we compared the cells isolated from ROs at early- (D60-90) and late-stages (D120 and afterwards) in detail. Although both were expandable, cells isolated from the late-stage ROs were the most optimal choice for the selection and enrichment of human MGCs, as they expanded to daughter MGCs with higher purity and expansion capability, and expressed typical MGC markers GS, which is consistent with the reported iMGCs expanded from ROs older than D160 (Couturier et al., 2021). In addition, Eastlake and colleagues reported that all of MGCs isolated from ROs aged at D34–D281 could express mRNA coding for the MGC markers CRALBP, GS, nestin and vimentin in two different passages (Eastlake et al., 2019). However, based on our results, cells from the early-stage ROs (D60–D90) did not express key MGC marker GS in protein level, and lost proliferative ability after six passages. According to the timecourse of iMGC development in ROs described above, we presumed that cells isolated from the early-stage ROs, which mainly consisted of RPCs or MGC precursors other than mature MGCs, were not able to continue differentiating or growing into mature MGCs under 2D culture conditions. Therefore, the late-stage ROs older than D120 were recommended to enrich iMGCs which recapitulated the features of primary human MGCs and were suitable for downstream investigation.

In the adult human retina, much controversy has arisen on the regeneration of MGCs, although it has been reported that MGCs have regenerative potential after retinal injury in the zebrafish, chick and mouse (Fischer and Reh, 2001; Raymond et al., 2006; Karl et al., 2008). Our current study along with previous reports demonstrated that the expanded iMGCs possessed the morphological, molecular and functional characteristics of primary human MGCs (Couturier et al., 2021). However, it remains unknown whether these iMGCs are capable of survival, regeneration or transdifferentiation *in vivo* or not. In 2019, Eastlake and colleagues intravitreally transplanted iMGCs into a rat model with NMDA-induced RGC depletion, and found that these iMGC grafts integrated into the retinal ganglion cell layer, leading to a partial rescue of the retinal ganglion cell function (Eastlake et al., 2019). In this study, we preliminarily explored the cell behavior of the expanded iMGCs after subretinal transplantation into the healthy NOD/SCID mice. Our results showed that the iMGCs expanded from the late-stage ROs could survive only for a short time and could not transdifferentiate into other types of retinal cells. HNA, a marker specific for human cells, was clearly expressed in the nucleus of the grated cells on 3 DAT, but in both nucleus and cytoplasm on 7 DAT, indicating the donor cells did not survive well, and started to deteriorate or apoptosis leading their nucleus to break down and spread out. Finally, these cells all disappeared on 21 DAT. Many factors contributing to the graft failure have been discussed in previous studies. For instance, the NOD/SCID mice without retinal defects in our current study could not provide specific microenvironments to promote the growth of donor cells, or the plasticity of the passaged iMGCs is limited. The passaged iMGCs growing in a 2D culture condition might have changed the characteristics of iMGCs in ROs which grow in a 3D culture environment mimicking human retina *in vivo*. Although immune rejection is believed to be one of the main causes of the graft failure (Singhal et al., 2008), it may not be the case since we used immunodeficiency NOD/SCID mice in our current study. However, we did not completely exclude this possibility since our immunostaining with anti-Iba1 antibody did show a certain degree activation of microglia following cell transplantation in NOD/SCID mice (data not shown). Therefore, substantial investigations will be needed to further evaluate the cell behavior of iMGCs *in vivo*, such as using a diseased mouse model, transplanting MGC precursors or MGCs directly isolated from hiPSC-derived ROs.

In conclusion, MGCs play important roles in physiological and pathological conditions in vertebrate retina, whose roles and related mechanisms still remain elusive in human retina due to the species differences, human tissue source limits and ethical concerns. Our results demonstrated that hiPSC-derived ROs could provide an accessible platform to directly study the developmental, morphological, molecular and functional characteristics of human MGCs. The simple approach and the optimal stage of ROs to enrich iMGCs have been established. Especially, iMGCs expanded from ROs lack of astrocyte contamination in comparison with those from human retinal tissue, which would serve as a better model and facilitate the follow-up investigation of MGC-related retinal disorders. This study also has limitations, such as only two hiPSC lines were evaluated and the expanded iMGCs did not survive well in the SRS of mouse retina. More studies are necessary to further explore the characteristics and applications of the enriched iMGCs.

## Data Availability Statement

The datasets presented in this study can be found in online repositories. The names of the repository/repositories and accession number(s) can be found below: https://www.ncbi.nlm.nih.gov/genbank/, GSE188698.

## Ethics Statement

The animal study was reviewed and approved by the Animal Ethics Committee of the Zhongshan Ophthalmic Center, Sun Yat-sen University.

## Author Contributions

RN, DZ, BX, GG, JX, PX, and YW performed the experiments and analyzed the data. RN and XZ designed the experiments. FP, BJ, JG, and XZ interpreted data. RN, DZ, and XZ wrote the manuscript. XZ conceived the study, supervised the project, secured the funds, and approved the manuscript. All authors contributed to the article and approved the submitted version.

## Funding

This work was supported by grants from the Science & Technology Project of Guangdong Province (2017B020230003), National Key Research and Development Program of the Ministry of Science and Technology (2017YFA0104101), National Natural Science Foundation of China (81970842, 82071265, and 82172957), and Science & Technology Project of Guangzhou (202102010288).

## Conflict of Interest

The authors declare that the research was conducted in the absence of any commercial or financial relationships that could be construed as a potential conflict of interest.

## Publisher's Note

All claims expressed in this article are solely those of the authors and do not necessarily represent those of their affiliated organizations, or those of the publisher, the editors and the reviewers. Any product that may be evaluated in this article, or claim that may be made by its manufacturer, is not guaranteed or endorsed by the publisher.
