# Supplementary material for: Spatial and Temporal Development of Müller Glial Cells in hiPSC-Derived Retinal Organoids Facilitates the Cell Enrichment and Transcriptome Analysis
Source: Front Cell Neurosci. 2022 May 19;16:820396. doi: 10.3389/fncel.2022.820396 (PMC9160306; doi:10.3389/fncel.2022.820396)
Supplement: Supplementary file 1 [file Data_Sheet_1.docx]

Supplementary Material


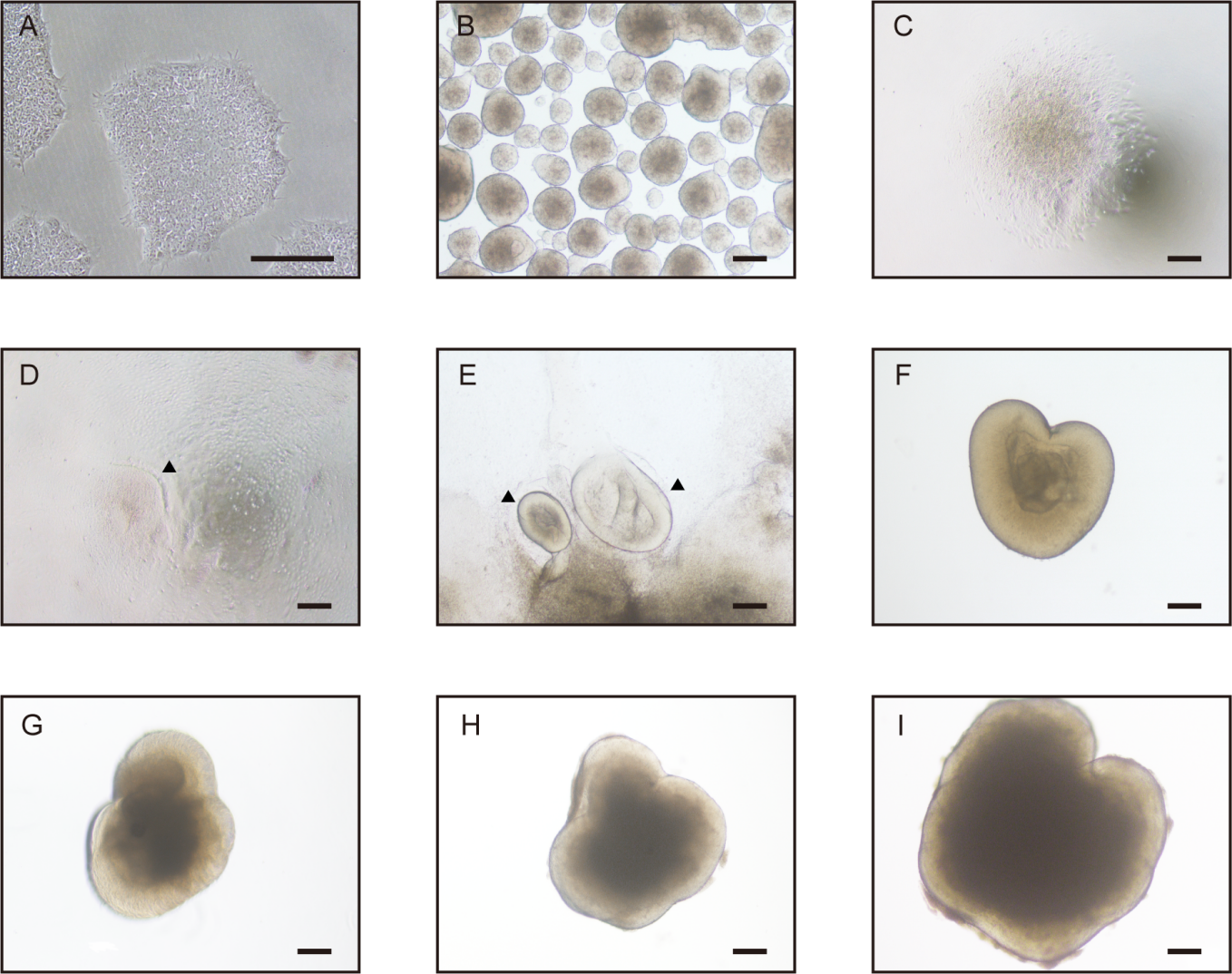


**Supplementary Figure 1.** Differentiation of hiPSCs into ROs. (A) hiPSCs. (B) hiPSC-derived EBs. (C) Plated EBs. (D) EFs (arrowhead). (E) OVs containing NR domains (arrowhead). (F) D30-RO. (G) D90-RO. (H) D120-RO. (I) D150-RO. hiPSCs: human induced pluripotent stem cells; ROs: retinal organoids; EBs: embryoid bodies; EFs: eye fields; OVs: optic vesicles; NR: neural retina. Scale bars = 200 μm (A–I).


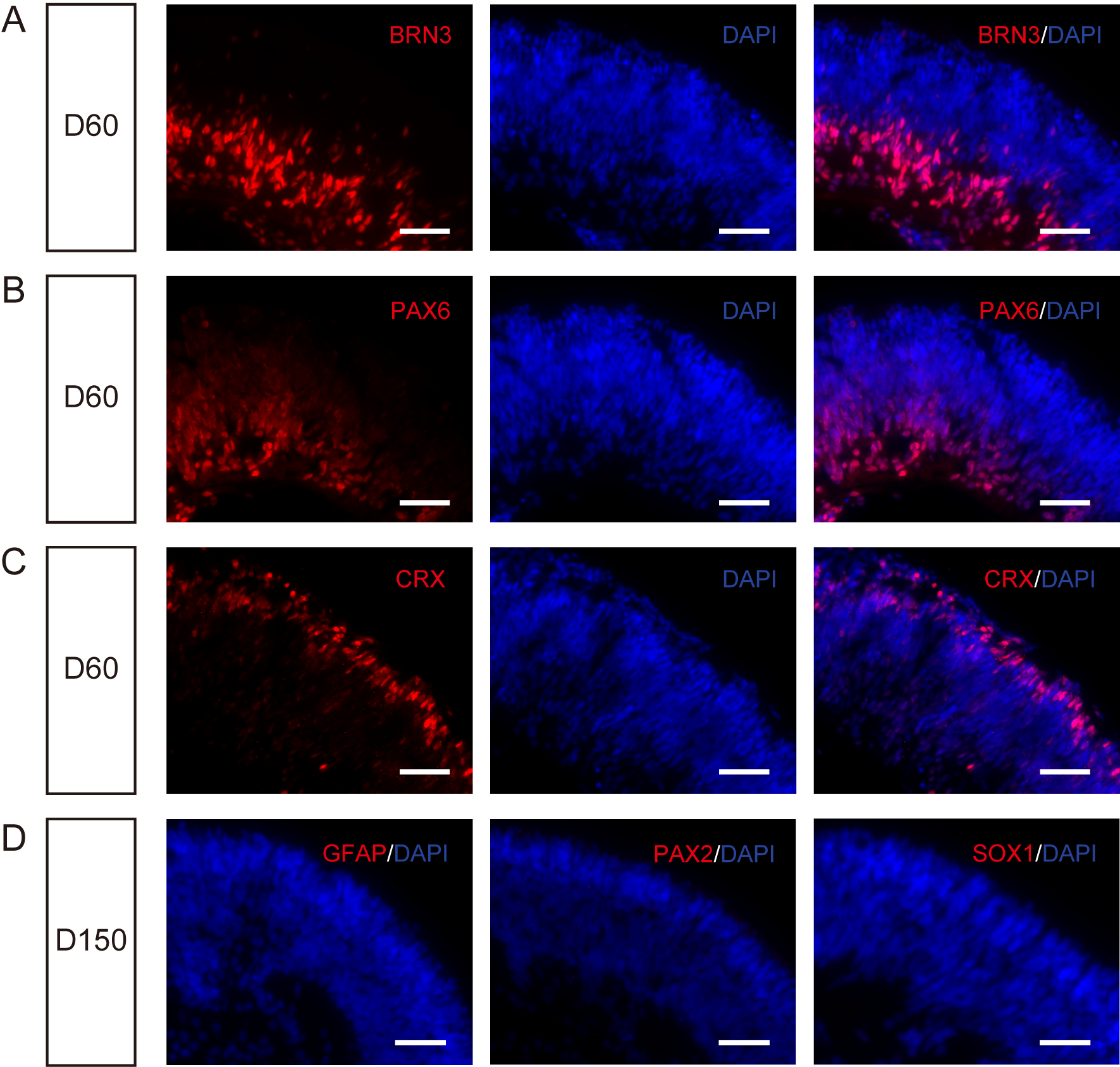


**Supplementary Figure 2.** Retinal cell types besides MGCs in hiPSC-derived ROs. (A–C) Immunofluorescence staining showed the expression of ganglion cell markers BRN3 and PAX6, and photoreceptor cell marker CRX in D60 ROs. (D) Immunofluorescence staining showed that astrocyte marker GFAP, optic stalk marker PAX2 and forebrain cell marker SOX1 were negative in the NR of ROs at D150. MGCs: Müller glial cells; hiPSCs: human induced pluripotent stem cells; NR: neural retina; ROs: retinal organoids. Scale bars= 50 μm (A–D).


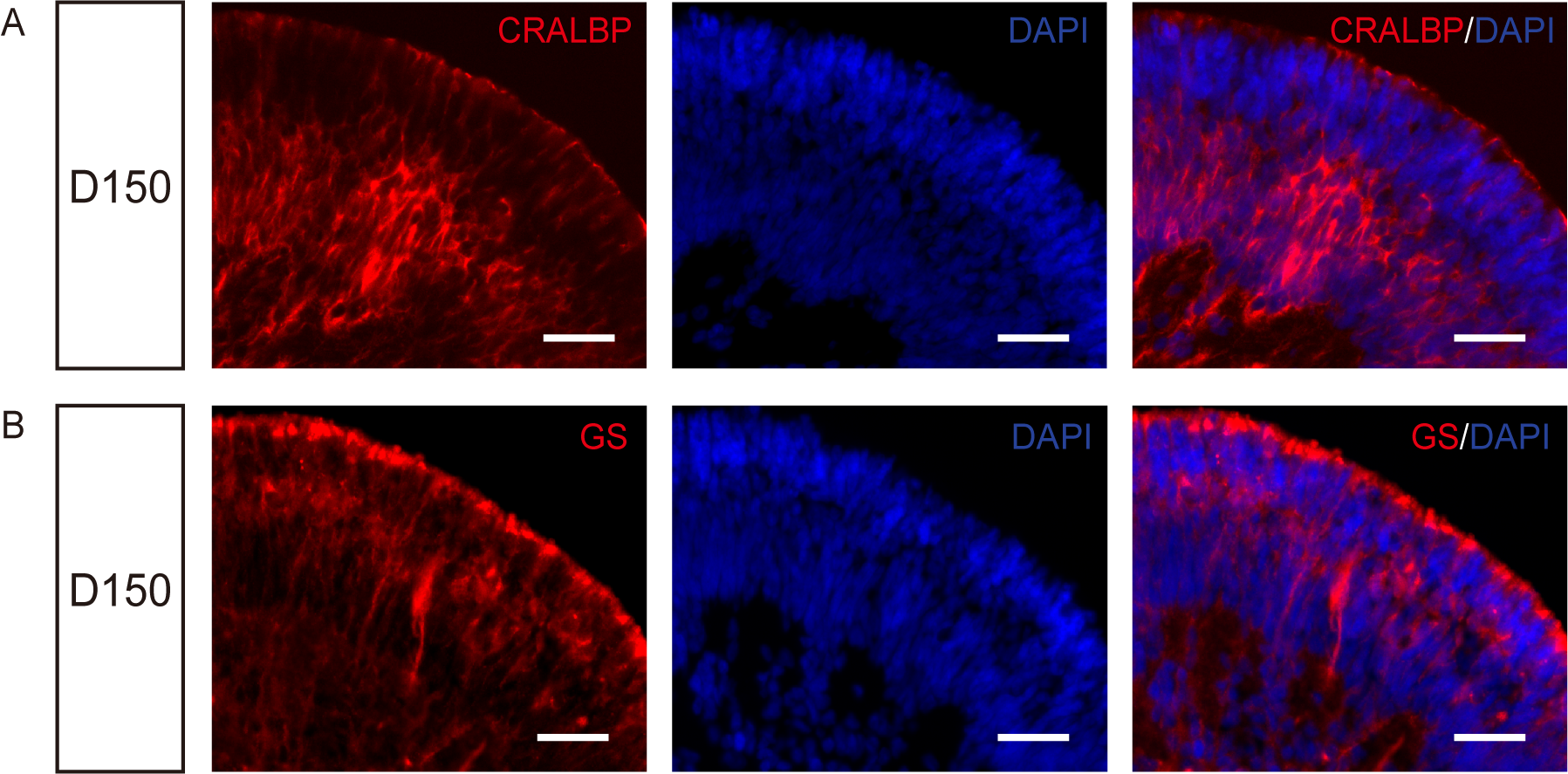


**Supplementary Figure 3.** Separated color channel images related to Figure 1B. (A–B) Immunofluorescence staining showed the expression of markers CRALBP and GS in D150-ROs. ROs: retinal organoids. Scale bars = 50 μm.


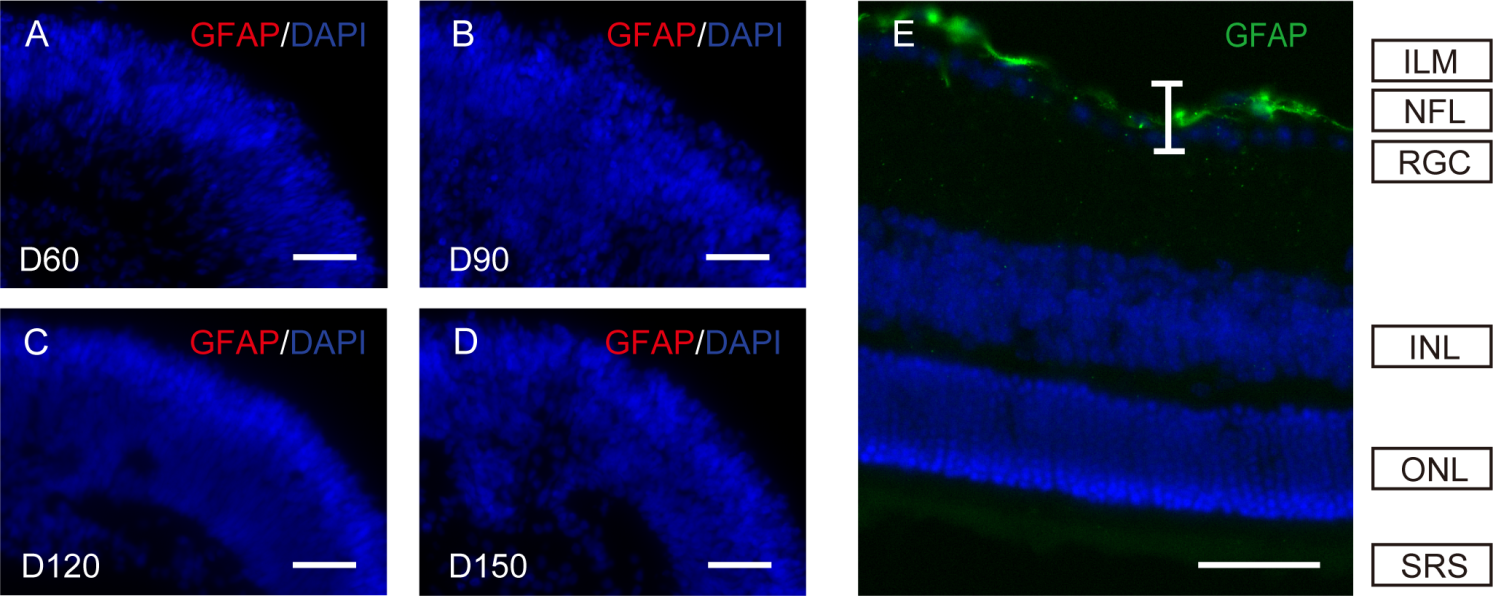


**Supplementary Figure 4.** The expression of GFAP in NR of ROs and in retina of adult NOD/SCID mouse shown by immunofluorescence staining. (A–D) At D60, D90, D120 and D150, there was no GFAP+ cells in the NR layer of ROs. (E) GFAP was specifically expressed in astrocytes located in the ILM or NFL of mouse retina and served as a parallel positive control, where was also adjacent to RGC. NR: neural retina; ROs: retinal organoids; RGC: retinal ganglion cell layer; INL: inner nuclear layer; ONL: outer nuclear layer; SRS: subretinal space; ILM: inner limiting membrane; NFL: nerve fiber layer. Scale bars= 50 μm.


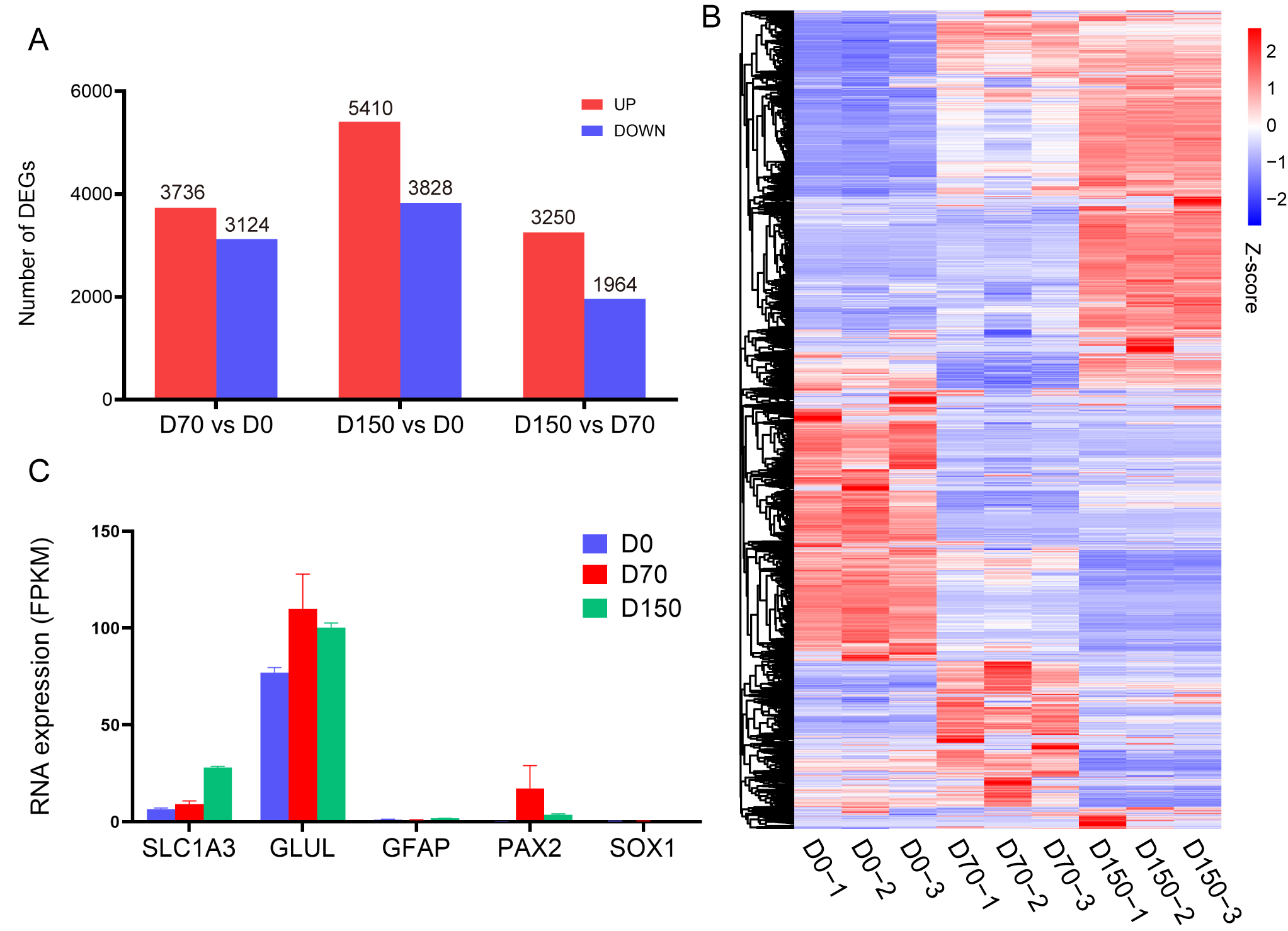


**Supplementary Figure 5.** RNA-seq analysis of MGC development in NR layers isolated from ROs at different stages. (A) Bar chart showed the number of the upregulated (UP) and downregulated (DOWN) differentially expressed genes (DEGs) among hiPSCs (D0), NR layers isolated from D70-ROs (D70) and D150-ROs (D150). (B) Heatmap showed all of the DEGs among hiPSCs and NR layers isolated from different-stage ROs. Blue to red indicated a gradient from low to high gene expression. (C) Bar chart exhibited the expression of *SLC1A3* (GLAST), *GLUL* (GS), *GFAP, PAX2* and *SOX1* among hiPSCs and NR layers isolated from different-stage ROs. hiPSC: human induced pluripotent stem cell; NR: neural retina; ROs: retinal organoids; -1, -2 and -3: experimental replicates; VS: versus. FPKM: a fragment per kilobase of transcript per million mapped reads.


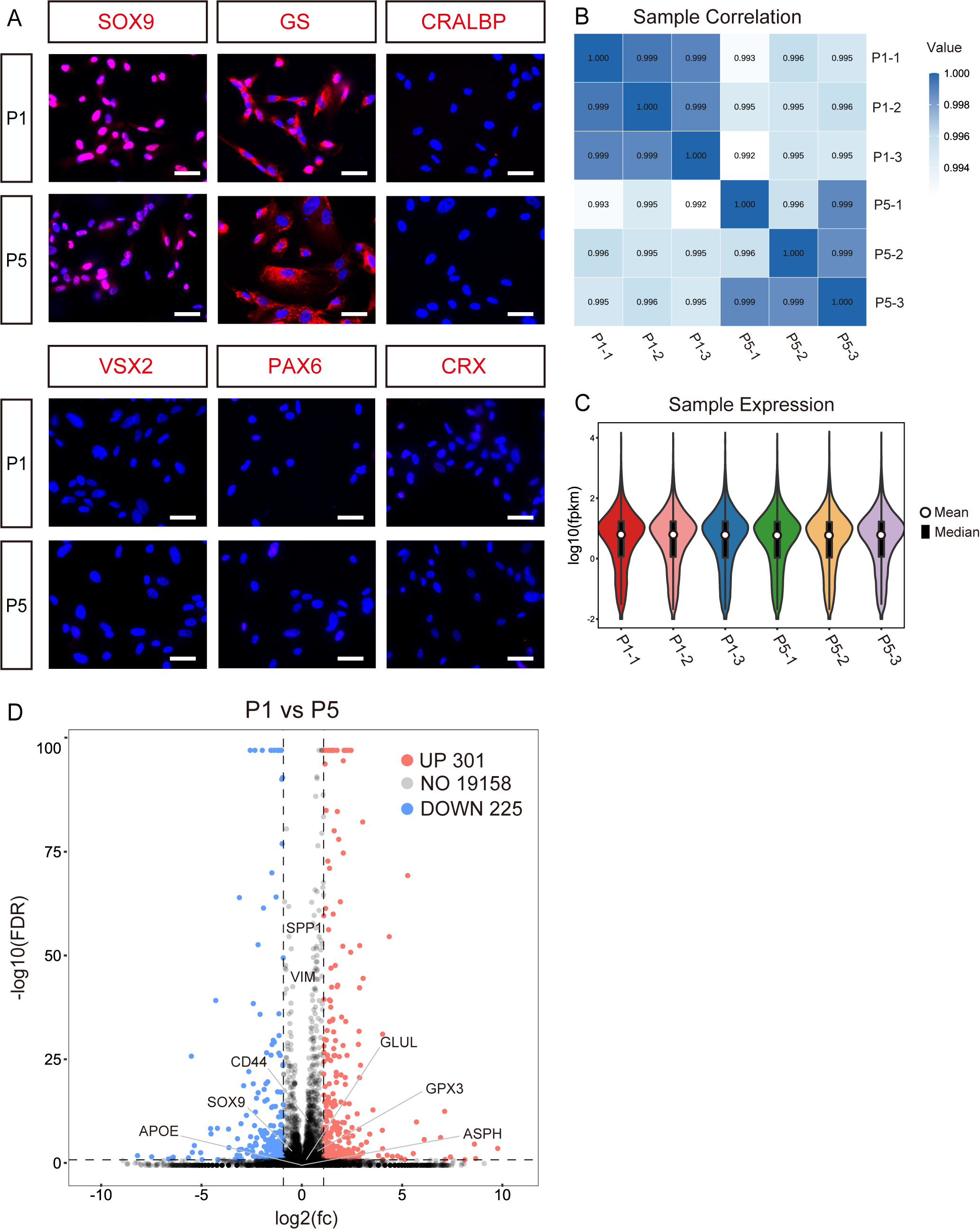


**Supplementary Figure 6.** The phenotype stability of iMGCs after serial passages. (A) Immunofluorescence staining showed the expression of markers SOX9, GS, CRALBP, VSX2, PAX6 and CRX in P1 and P5 iMGCs. (B) Heatmap showed the sample correlation of the P1 and P5 iMGCs. (C) Violin plot analysis showed the expression levels and distributions of all genes in P1 and P5 iMGCs. (D) Volcano plot exhibited that there is no difference in the expression of classical MGC-related genes between P1 and P5 iMGCs. -1, -2 and -3: experimental replicates; P1 and P5: passage 1 and 5; FDR: false discovery rate; FC: fold change; UP: upregulation; DOWN: downregulation; NO: no significance; VS: versus; fpkm: fragment per kilobase of transcript per million mapped reads; iMGCs: induced Müller glial cells. Scale bars = 50 μm (A).


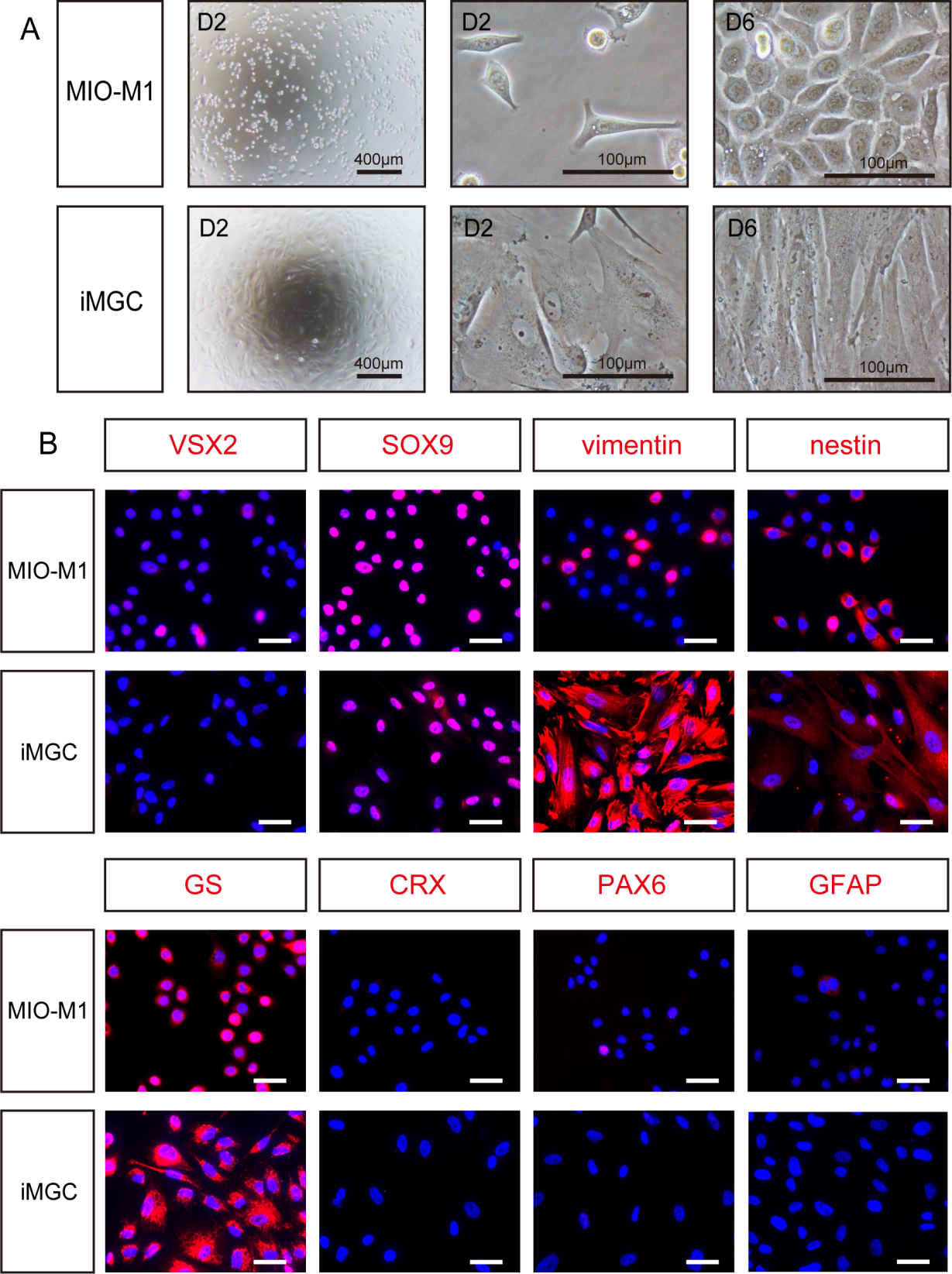


**Supplementary Figure 7.** The comparison of MIO-M1 line and iMGCs expanded from hiPSC-derived ROs in morphology and markers expression. (A) Bright-field images showed the distinguished morphology of the MIO-M1 and iMGCs. (B) Immunofluorescence staining showed the expression of markers VSX2, SOX9, vimentin, nestin, GS, CRX, PAX6 and GFAP in the MIO-M1 and iMGCs. iMGC: induced Müller glial cells. Scale bars = 50  μm (B).

**Supplementary Table S1.** Primary Antibodies Used for Immunofluorescence Staining

| Antigen | Host | Dilution Ratio | Catalog Number | Supplier |
| --- | --- | --- | --- | --- |
| VSX2 | sheep | 1/200 | ab9016 | Millipore |
| Ki67 | mouse | 1/200 | 550609 | BD |
| SOX9 | rabbit | 1/250 | A19710 | Abclonal |
| vimentin | rabbit | 1/50 | BM4029 | Boster |
| nestin | rabbit | 1/200 | ab82375 | Abcam |
| CRALBP | mouse | 1/500 | ab15051 | Abcam |
| GS | mouse | 1/200 | 610517 | BD |
| PAX6 | mouse | 1/50 | 528427 | DSHB |
| CRX | mouse | 1/800 | H00001406-M02 | Abnova |
| BRN3 | goat | 1/100 | sc-6026 | Santa Cruz |
| GFAP | rabbit | 1/50 | BM4287 | Boster |
| HNA | mouse | 1/400 | ab191181 | Abcam |
| PAX2 | rabbit | 1/100 | A3067 | Abclonal |
| SOX1 | rabbit | 1/50 | BM4661 | Boster |
